# Exploring the Possibility of Photoplethysmography-Based Human Activity Recognition Using Convolutional Neural Networks

**DOI:** 10.3390/s24051610

**Published:** 2024-03-01

**Authors:** Semin Ryu, Suyeon Yun, Sunghan Lee, In cheol Jeong

**Affiliations:** 1 Department of Artificial Intelligence Convergence, Hallym University, Chuncheon 24252, Republic of Korea; sr@hallym.ac.kr (S.R.); syyun@hallym.ac.kr (S.Y.); 2 Cerebrovascular Disease Research Center, Hallym University, Chuncheon 24252, Republic of Korea; sh.lee@hallym.ac.kr; 3 Department of Population Health Science and Policy, Icahn School of Medicine at Mount Sinai, New York, NY 10029, USA

**Keywords:** human activity recognition, photoplethysmography, window size, convolutional neural networks, cross-subject validation

## Abstract

Various sensing modalities, including external and internal sensors, have been employed in research on human activity recognition (HAR). Among these, internal sensors, particularly wearable technologies, hold significant promise due to their lightweight nature and simplicity. Recently, HAR techniques leveraging wearable biometric signals, such as electrocardiography (ECG) and photoplethysmography (PPG), have been proposed using publicly available datasets. However, to facilitate broader practical applications, a more extensive analysis based on larger databases with cross-subject validation is required. In pursuit of this objective, we initially gathered PPG signals from 40 participants engaged in five common daily activities. Subsequently, we evaluated the feasibility of classifying these activities using deep learning architecture. The model’s performance was assessed in terms of accuracy, precision, recall, and F-1 measure via cross-subject cross-validation (CV). The proposed method successfully distinguished the five activities considered, with an average test accuracy of 95.14%. Furthermore, we recommend an optimal window size based on a comprehensive evaluation of performance relative to the input signal length. These findings confirm the potential for practical HAR applications based on PPG and indicate its prospective extension to various domains, such as healthcare or fitness applications, by concurrently analyzing behavioral and health data through a single biometric signal.

## 1. Introduction

Human activity recognition (HAR) entails automatically detecting the various daily physical activities individuals perform. These activities can be captured using an array of devices, such as cameras or motion, physiological, acoustic, and ambient (including infrared and magnetic) sensors [1].

Depending on the sensing method employed, HAR can be broadly categorized into external and internal sensor-based approaches. External methods encompass optical signals (video), Wi-Fi signals (utilized in efficient Wi-Fi-based HAR), environmental signals (e.g., smart home data, including temperature, humidity, ${\mathrm{CO}}_{2}$ levels, light intensity), and even seismic waves [2,3,4,5,6]. Notably, camera-based approaches have demonstrated remarkable performance in HAR, particularly with advancements in artificial neural networks [7,8,9]. However, due to privacy concerns associated with camera-based systems, alternative approaches utilizing different types of sensors have emerged. With the proliferation of smart devices, wearable sensors have garnered significant attention for addressing privacy and security concerns [10,11,12,13,14].

Among internal sensing methods, studies employing inertial measurement units (IMUs) have been prevalent, owing to their inherent capability to directly capture signals related to kinematics. In recent HAR and other human–computer interaction studies, signals received by biomechanical sensors, such as IMUs, are also classified as physiological/biological/biomedical/biometric signals [15,16]. Additionally, biomechanical sensors suitable for HAR include electrogoniometers (EGMs) and electronic protractors such as those used to record electromyograms (EMGs) or monitor galvanic skin response (GSR)/electrodermal activity (EDA). These sensors measure joint angles or electrical activity generated by skeletal muscles, facilitating the classification of daily activities [17,18,19]. Alternatively, the increasing focus on healthcare devices has led to a gradual rise in proposals for HAR systems based on biometric signals such as electrocardiography (ECG) and photoplethysmography (PPG). ECG detects changes in electrical characteristics that occur during the cardiac cycle [20]. Wearable devices capable of running ECG hold significant potential for HAR applications but necessitate disposable electrodes, resulting in inconvenience and additional costs. PPG serves as an alternative for measuring heart rate and cardiovascular rhythm, detecting alterations in light absorption by vascular tissue as blood flow changes due to the cardiac cycle [21]. It is highly accessible as it can be measured using pulse oximeter sensors embedded in many off-the-shelf, wearable devices, such as smartwatches [22]. However, to date, PPG signals have been sparingly utilized for HAR systems, often as a supplement to IMU or ECG signals [23].

Biometric signals inherently contain information about an individual’s health. If PPG signals prove suitable for HAR, they can be applied across various domains, as health and behavioral data can be simultaneously analyzed from a single sensor. PPG sensors can be manufactured in very compact sizes and can be measured at the fingertip, allowing for integration into wearable devices like smartwatches or rings, thereby minimizing user inconvenience during signal measurement. Additionally, compared to IMU sensors, PPG can reflect cardiac signals. Thus, PPG holds advantages for extending applications in health and medical fields, such as biometric authentication, patient monitoring, and fall detection. For instance, a recent study utilizing finger PPG reconstructed ambulatory blood pressure (ABP) for further medical applications [24,25]. To facilitate the practical application of PPG, we herein propose and evaluate an HAR system based on PPG signals newly collected from 40 participants while performing daily activities. The collected data underwent pre-processing and classification by an end-to-end model based on a one-dimensional convolutional neural network (1D CNN). Performance evaluation was conducted through cross-subject CV to ensure generalizability and mitigate inflated results. Furthermore, we investigated the optimal window size by assessing performance relative to the input PPG signal length. The results suggest that the proposed approach can facilitate real-world implementation of practical HAR systems.

## 2. Related Work

Numerous IMU-based methods have been investigated to recognize human activity. Pesenti et al. [26] presented a deep learning-based approach utilizing IMUs for industrial exoskeleton robots. The method utilized long short-term memory (LSTM) networks to classify human activities and payload, classifying five behavior and interaction types with an accuracy of 90.8%. Li et al. [27] proposed a deep learning model combining ResNet and BiLSTM, which effectively extracts the spatial and temporal features of sensor data. They constructed their dataset by collecting activity data from a custom-built IMU module mounted on a human leg. Kim et al. [28] employed Conformer, a state-of-the-art model in speech recognition, to improve HAR performance. The Conformer outperformed the baseline models, Transformer and a 1D CNN, achieving an accuracy of 98.1%, 99.7%, and 99.3% on WISDM, PAMAP2, and UCI-HAR datasets, respectively. Jaramillo et al. [29] proposed a novel human activity prediction system, HAP, based on forecasted IMU signals. The HAP system employed a prediction model based on attention and sequence-to-sequence architecture and a pre-trained Bi-LSTM model to predict future activity from IMU data. The model achieved an accuracy of 97.96% on the PAMAP2 dataset. Challa et al. [30] developed an optimized deep learning model that classifies human activities captured by IMUs. Their model combined convolutional layers and Bi-LSTM units to extract spatial and temporal features. The model was evaluated on PAMAP2, UCI-HAR, and MHEALTH datasets with accuracies of 94.91%, 97.16%, and 99.25%, respectively.

Some research groups have proposed biometric sensor-based HAR approaches. Brophy et al. [31] applied a machine vision approach for HAR based on PPG signals to predict activities and achieved 75.8% accuracy on the Wrist PPG During Exercise dataset [32]. The result demonstrates the feasibility of implementing an optical sensor-based solution for HAR and heart rate monitoring systems. Muhmud et al. [33] proposed a multi-layer LSTM-based deep neural network that integrates multimodal features from multiple sensors for HAR. They used the Wrist PPG During Exercise [32] dataset, and the resulting accuracy was 74.7% and 72.1% for IMU and PPG sensors, respectively. Almanifi et al. [22] investigated the potential of using PPG sensors in HAR systems as an alternative to ECG sensors by comparing the resulting performance using ECG and PPG signals. An ensemble of pre-trained models such as Resnet50V2, MobileNetV2, and Xception were used to classify four activity types based on PPG signals measured at the wrist; the results were compared with those using an ECG-based approach. The classification accuracy was 88.91% and 94.28% for PPG and ECG, respectively, suggesting the feasibility of using PPG sensors in situations where ECG sensors are unavailable. Hnoohom et al. [23] proposed PPG-NeXt, an innovative deep learning method to extract relevant features from PPG signals and generate predictions. The PPG-NeXt model was validated on three benchmark datasets and achieved an F-1 measure of over 90%.

In summary, IMU-based approaches have been intensively investigated in the HAR field and have achieved better performance than biometric signal-based methods. Recently, methods based on ECG and PPG signals have gained interest; however, most studies have been conducted on a few public datasets with limited subjects. For practical applications, analysis utilizing a larger database is required. In addition, the performance should be evaluated based on cross-subject CV to ensure scalability and avoid inflated results [34].

## 3. Method

**Figure 1:**
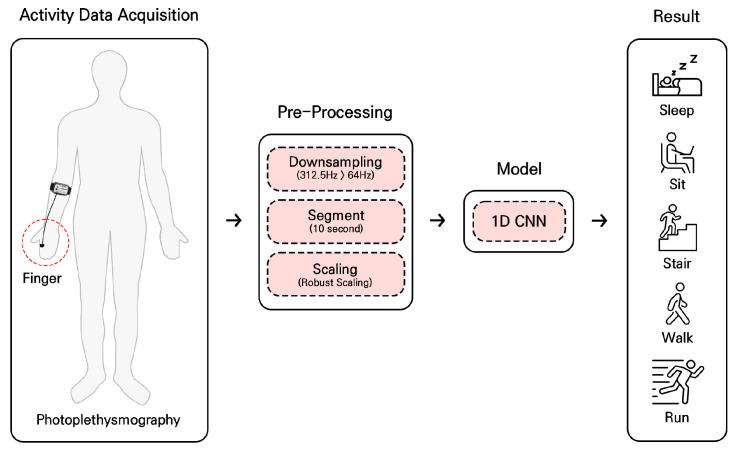
Overview of the proposed human activity recognition (HAR) framework based on photoplethysmogram signals.

Figure 1 depicts the overall structure of the proposed HAR framework based on PPG signals. Raw PPG measurements from each participant are downsampled, segmented, and re-scaled to be used as the input representation for the proposed 1D CNN model. The model then classifies the input data into five daily activities: sleeping, sitting (working), ascending and descending stairs, walking, and running. The details are presented in the following sections.

### 3.1. Data Description

Table 1 summarizes information on the subjects enrolled in this study. Forty healthy participants (twenty males) ages 19 or older (average 23.95 years) were involved in the study. Before the experiment, we explained the experimental procedure to all participants and collected their informed consent and demographic information, such as age, gender, height, and weight. All procedures followed the guidelines approved by the Institutional Review Board of Hallym University (HIRB-2022-025).

**Figure 2:**
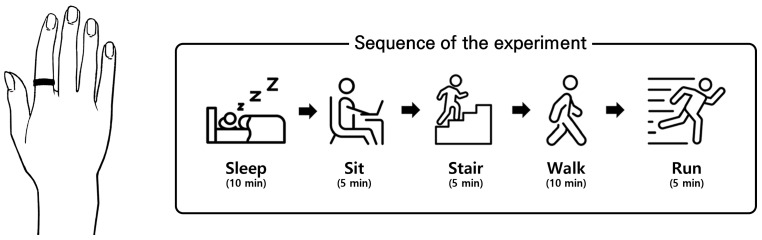
Sequence of the experiment. Participants performed five activities wearing the PPG sensor on their index finger.

Figure 2 depicts the experimental procedure. All data were collected using a commercial data-acquisition system (MP150, BIOPAC Systems Inc., CA, USA) with a wireless PPG module (BN-PPGED, BIOPAC Systems Inc., CA, USA). The raw PPG signals were sampled at 312.5 Hz. The protocol included five activities (sleeping, sitting (working), ascending and descending stairs, walking, and running) commonly performed in everyday life. The participants were instructed to perform these activities while wearing the PPG module on their index finger, as follows:

- Sleeping: Subjects laid on a mat with their eyes closed for 10 min with minimal movement.
- Sitting (working): This activity was included to replicate sitting at a desk and working. Subjects sat still in a chair and performed work-related tasks, such as using a computer or reading a book, for 5 min.
- Ascending and descending stairs: Subjects walked up and down stairs for 5 min, without any restrictions on speed of step or arm movements.
- Walking: Subjects walked on a treadmill for 5 min at approximately 5–6 km/h without any restrictions on arm movements. This speed was chosen based on [35], which examined the walking and running speeds of 230 people ages 20–79.
- Running: Subjects ran on a treadmill for 5 min at approximately 8 km/h without any restrictions on arm movements. This speed was also selected based on [35]. The subjects were instructed to include a flight phase (the time in the running gait cycle when both feet are in the air and the body is no longer in contact with the ground) during the run to distinguish it from walking. Participants were given sufficient breaks after each session to stabilize their heart rate.

### 3.2. Pre-Processing

**Figure 3:**
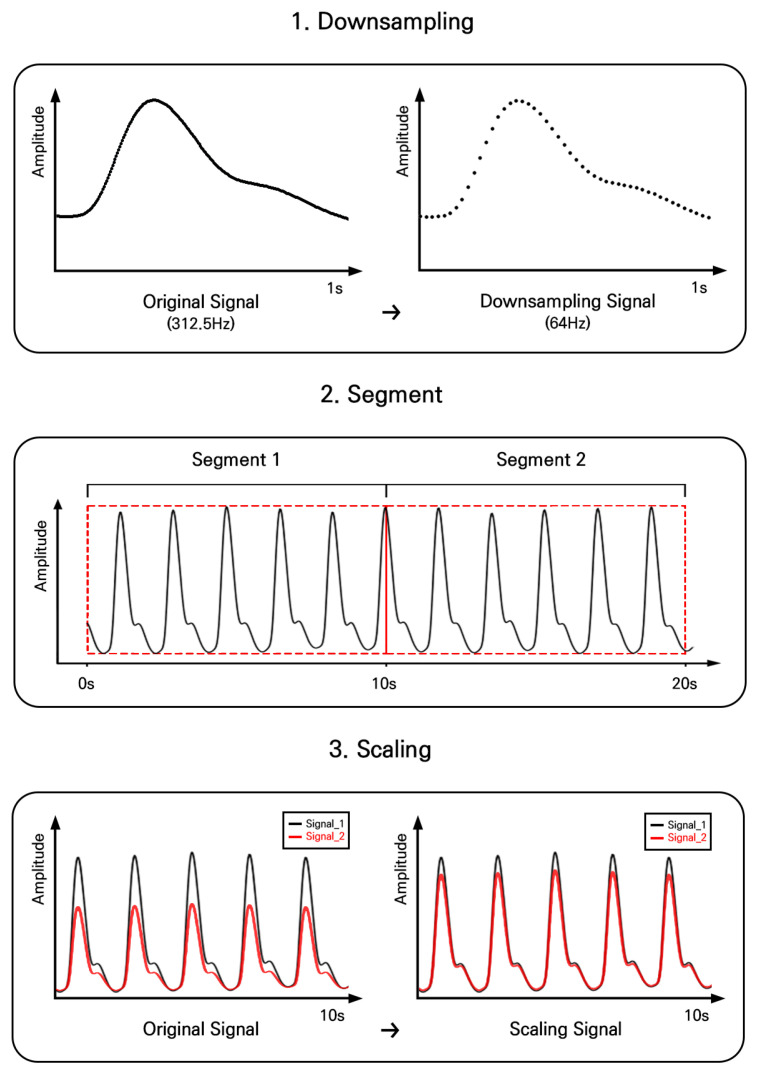
Pre-processing procedure, including downsampling, segmentation, and re-scaling with example data.

Figure 3 shows the entire pre-processing procedure with an example from the collected samples. The raw PPG signal was pre-processed in three steps: downsampling, segmentation, and re-scaling. First, the raw signal (originally acquired at 312.5 Hz) was downsampled to 64 Hz, the lowest rate from the public PPG dataset [36]. Then, the downsampled signal was segmented without overlap. Finally, the signal amplitude was standardized using the median and interquartile range, i.e., robust scaling, to constrain the effect of outliers [37]. The pre-processed signal was used as the input representation for the proposed deep learning architecture.

### 3.3. Model

**Figure 4:**
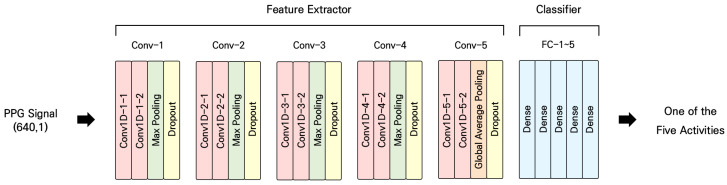
Structure of the proposed network.

Given their capacity to learn both local and global features from time-series data, CNNs have been extensively employed in HAR applications [38,39,40,41,42]. In this study, we adopted a deep learning architecture based on a 1D CNN to predict five daily activities by learning intrinsic features based on PPG signals. Figure 4 schematically describes the proposed deep learning model. The pre-processed PPG signal was used as the input representation. The model comprised ten convolutional layers and four max-pooling layers, with pooling size 2. For the convolutional layers, the number of filters was 64, 64, 128, 128, 256, 256, 512, 512, 1024, and 1024. The kernel size was 5 for the first two layers and 3 for the rest; the stride was 1 for all convolutional layers. A leaky rectified linear unit (Leaky ReLU) was used as the activation function, except at the output node that used softmax activation. A global average pooling layer was applied to convert the feature map extracted from the convolutional layers to a 1D vector. This vector passed through five fully connected layers, with 512, 256, 128, 64, and 5 nodes; it was then softmax-activated to generate a prediction. A dropout was applied after the pooling layers to prevent over-fitting. We tried to simplify the model with the least performance drop as we plan to implement the proposed system in an embedded environment in the future.

### 3.4. Experiment

**Figure 5:**
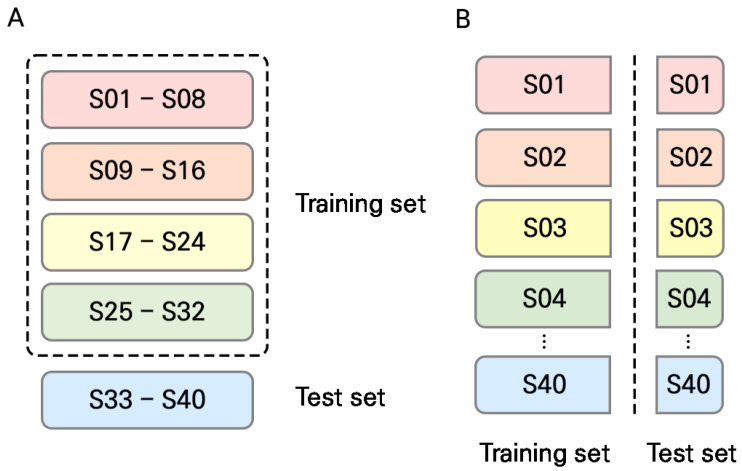
The dataset was divided into five groups for both the (**A**) cross- and (**B**) intra-subject CV.

A performance evaluation using the subjects’ data not utilized in the training phase is required to ensure practical applicability. To this end, in experiment I, a cross-subject CV scheme was used to evaluate the generalization performance of the proposed approach. We divided the entire dataset into five groups (or folds), each containing eight subjects, as shown in Figure 5A. In other words, 32 subjects’ data were used to train the model, while the remaining were used to assess the model’s performance. An intra-subject CV was also conducted for comparative analysis, as shown in Figure 5B. The number of training and testing data points for each model were 5760 and 1440, respectively. This procedure was repeated for each fold, yielding five models. The performance of each model was evaluated in terms of classification accuracy. In this experiment, the window size was fixed at 10 s.

The effect of the input signal length on the HAR system performance has been investigated to determine the “optimal” or “cut-off” window size [43,44]. The optimal window size significantly varies according to parameters such as signal type, number of class categories, and activity type [45,46]. Therefore, in experiment II, we investigated the trade-off between the window size and performance by varying the window size from 2 to 20 s.

## 4. Results

**Figure 6:**
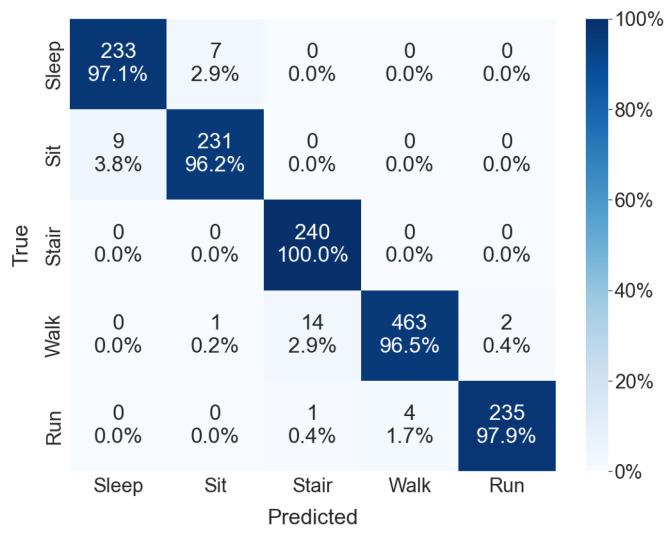
Normalized confusion matrix for the cross-subject CV of test fold 2 in experiment I. The rows and columns correspond to the actual and predicted class labels, respectively.

Table 2 presents the results of intra-subject and cross-subject CVs from experiment I in terms of accuracy, precision, recall, and F-1 measure. All metrics, except accuracy, are reported as weighted averages. The observed performance showed minimal variance across different folds. The test accuracies (mean ± standard deviation) for the intra- and cross-subject CV were 98.6 ± 0.49% and 95.1 ± 1.6%, respectively. This outcome underscores the robustness of the proposed model, which consistently achieved above 92% accuracy across all test folds in cross-subject CV. Further investigation into classification performance differences among class categories (activities) was conducted. As shown in Table 3, the proposed model demonstrated well-balanced performance across all classes. Figure 6 depicts the normalized confusion matrix. Predominant misclassifications were observed between sleeping and sitting, followed by those between ascending/descending stairs and walking. Nonetheless, accuracy remained above 96% for all classes.

**Figure 7:**
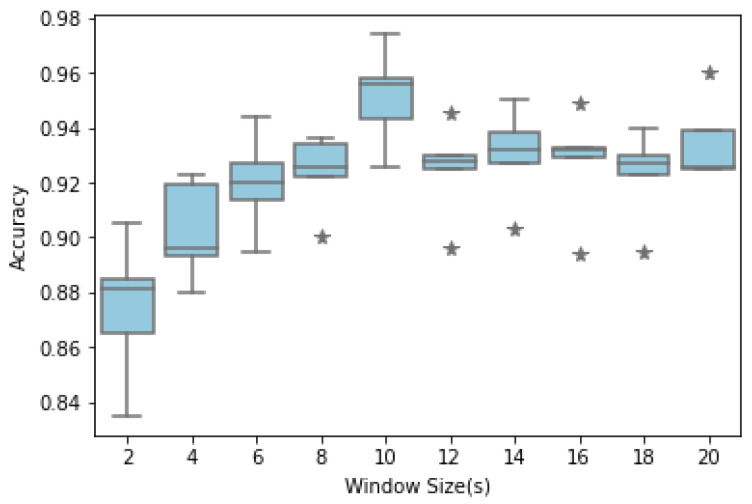
Performance comparison box plot between different window sizes. The asterisk (*) signifies the outliers.

In experiment II, we examined different fixed window sizes from 2 to 20 s in increments of 2 s for all test folds. Figure 7 shows changes in the model’s performance as a function of window size. The test accuracy gradually increases with increasing window sizes and stabilizes after the window size reaches 10 s. The model achieves reasonable performance even for small window sizes, reaching accuracies of 87.42% and 90.22% at 2 and 4 s, respectively.

## 5. Discussion

### 5.1. General Discussion

We performed cross-subject CV to assess the generalization ability of the proposed model. The model could sense the type of activities remarkably well, even for data not used for the training phase, i.e., blind test data. However, as shown in Figure 6, some misclassified classes exist. Most misclassifications are observed between the sleeping and working classes. These two activities have similar exercise intensity as they do not involve considerable physical movement compared to other activities. The second-highest misclassifications are between the ascending/descending stairs and walking classes. For ascending/descending stairs, both feet are crossed while performing the activity, with at least one foot supporting the ground. It is kinematically similar to the walking mechanism, confusing the proposed model between the two activities. Nonetheless, the resulting accuracy was above 96% for all classes, demonstrating that the proposed approach supports real-world applications.

For the biometric signals, a larger window size implies that more than one period of cardiac activity can be captured in a single window. Thus, more features, such as heartbeat, can be learned. However, an increase in window size did not necessarily increase accuracy [44,45,46]. To determine the optimal input signal length, i.e., window size, we segmented the PPG signal into lengths ranging from 2 to 20 s in increments of 2 s. The accuracy increased with increasing window sizes but converged drastically as the window size reached 10 s or more. Thus, the optimal window size was decided to be approximately 10 s. This suggests that window sizes above a certain length that already contain sufficient features are unnecessary. Notably, the model achieved acceptable accuracy even for small window sizes (e.g., 2 and 4 s).

However, recent studies have also suggested the use of longer window lengths, with durations measured in minutes or hours rather than seconds, which may be more suitable for prolonged activities such as sleep [45]. Therefore, in future research, it is imperative to consider longer window sizes alongside acquiring adequate data to facilitate 24 h monitoring and auto-labeling. Consequently, the selection of the appropriate window size is expected to be contingent upon the type of hardware and computational capacity available.

Table 4 provides a review of recent research on HAR, including the proposed approach. Although there have been a growing number of biometric signal-based studies, there are relatively few compared to IMU-based studies; moreover, these studies rely on limited datasets. The number of subjects in the studies ranged from 7 to 15; a larger database would be needed to derive more generalized results. Furthermore, only a few studies explicitly performed cross-subject CV, as shown in bold with an asterisk. A correct CV method should be employed to avoid the inflated result caused by data leakage [34]. In this study, we evaluated the proposed model through cross-subject CV on 40 subjects and achieved an average accuracy of 95.14%. To ensure fairness in comparison, we pursued two avenues: (1) applying models used in similar studies (PPG-NeXt and LSTM) to our dataset, and (2) employing the proposed deep learning architecture on another dataset (PPG-Dalia, PPG-ACC, and Wrist PPG During Exercise). As illustrated in Table 4, a substantial performance gap was observed between cross-subject and intra-subject validation, with the proposed model notably outperforming other models, particularly in cross-subject CV. Furthermore, it was noted that the proposed model demonstrates superior performance on larger datasets compared to smaller ones. These results underscore the necessity for large datasets and cross-subject CV, providing crucial guidance for future biometric signal-based HAR studies.

### 5.2. Limitations and Future Work

The participants involved in this study were healthy university students with an average age of 22.9. As different age groups have different motor abilities, other features could be captured even in the same activity. Therefore, a model trained with only data from a certain age group might produce biased results. In addition, people with underlying health issues would have distinctly different behavioral patterns than those of a healthy person. Therefore, constructing a dataset including participants from various age groups and health conditions is necessary to generate a more practical model.

IMU-based HAR studies have attempted to recognize up to 18 activity classes, whereas biometric signal-based studies have been conducted for 4–8 classes. The activities dealt with in biometric signal-based studies include sitting, playing soccer, cycling, driving, eating, and walking. Our study protocol included five activities: sleeping, sitting, ascending/descending stairs, walking, and running. Most of these activities are expected to be categorized well because each has distinct kinematic characteristics. However, it would be worth evaluating whether the proposed model can classify other activities. More sophisticated algorithms may be required for fine-grained activity classification, such as watching TV, working on a computer, or reading a book.

Additionally, before our work, the performance of PPG-based HARs was generally lower than that of IMU-based HARs. Although our results suggest possibilities, they still need to be improved. Above all, PPG-based HARs have yet to provide superior results for similar or complex behaviors [23,33,44]. Future research should investigate whether PPG-based HAR can detect activities with either similar characteristics across classes or more complex characteristics.

The main purpose of this study was to explore the feasibility of a PPG-based HAR system. Although we achieved reasonable performance with the proposed model, further evaluation through different architectures is needed to find an optimal model. Moreover, methods for reducing the feature space and designing high-level features should also be considered [52,53,54]. Consequently, it is imperative to explore the trade-off between performance and computational cost by applying classical methods such as feature space reduction and high-level feature design, as well as recent deep learning-based techniques.

The aforementioned aspects would hinder the potential application of the proposed approach. In the future, we plan to (1) involve more participants from different age groups and health conditions, (2) increase the number of activities for classification, and (3) comprehensively evaluate performance with various artificial intelligence models.

## 6. Conclusions

We proposed a PPG-based HAR system and evaluated the feasibility of the proposed system to be extended to real-world applications. First, we constructed a new dataset based on the PPG signals collected from 40 participants during their daily activities. Subsequently, a 1D CNN model was designed to classify five different activities. The model’s performance was evaluated in terms of test accuracy, precision, recall, and F-1 measure. The experimental results validated the feasibility of the proposed approach, achieving an average test accuracy of 95.14% in cross-subject CV. Furthermore, by comprehensively assessing the performance with respect to the input signal length, we found the optimal window size to be 10 s. The results demonstrated the potential use of the PPG-based HAR system in practical applications. We expect the proposed approach to be extended to several domains, such as healthcare or fitness applications, by simultaneously analyzing behavioral and health information from a single biometric signal.

## Figures and Tables

**Table 1:** Demographic information of the participants: BMI, body mass index; SD, standard deviation.

		Age	Height (cm)	Weight (kg)	BMI
Female	Mean	23.7	161.4	54.7	20.9
( *n* = 20)	SD	2.5	5.2	9.2	2.8
Male	Mean	24.3	175.0	72.8	23.8
(*n* = 20)	SD	2.7	6.0	9.5	3.1
ALL	Mean	24.0	168.2	63.7	22.4
(*n* = 40)	SD	2.6	8.8	13.0	3.3

**Table 2:** Intra- and cross-subject test metrics (SD: standard deviation).

Experiment	Fold	1	2	3	4	5	Mean	SD
Intra-subject	Accuracy	0.98	0.99	0.99	0.99	0.98	0.99	0.005
	Precision	0.98	0.99	0.99	0.98	0.98	0.98	0.005
	Recall	0.98	0.99	0.99	0.99	0.98	0.99	0.005
	F-1 measure	0.98	0.99	0.99	0.99	0.98	0.99	0.005
Cross-subject	Accuracy	0.96	0.97	0.94	0.96	0.93	0.95	0.015
	Precision	0.95	0.97	0.93	0.95	0.92	0.94	0.017
	Recall	0.96	0.98	0.94	0.95	0.92	0.95	0.020
	F-1 measure	0.96	0.97	0.94	0.95	0.92	0.95	0.017

**Table 3:** Precision, recall, and F-1 measure for test fold 2 in experiment I: cross-subject CV.

Class	Precision	Recall	F-1 Measure
Sleep	0.96	0.97	0.97
Sit	0.97	0.96	0.96
Stair	0.94	1.00	0.97
Walk	0.99	0.96	0.98
Run	0.99	0.98	0.99
Average	0.97	0.97	0.97

**Table 4:** Descriptions and results of recent studies on HAR. The asterisks indicate that the results were evaluated through cross-subject CV.

Signal	Paper	Data	Subject	Class	Model	Performance
IMU	Arani et al. [44], 2021	PPG-DaLiA [36]	15	5	Random Forest	F1-Score 94.07% (10-fold)
						**F1-Score 83.16%** **(Leave-One-Subject-Out)**
	Mahmud et al. [33], 2020	Wrist PPG During Exercise [32]	8	4	LSTM	Accuracy 74.7%
	Li et al. [27], 2022	Private dataset	5	6	ResNet + BiLSTM	Accuracy 96.95%
		WISDM [47]	36	6		Accuracy 97.32%
		PAMAP2 [48]	9	18		**Accuracy 97.15%** **(Cross-subject)**
	Kim et al. [28], 2022	WISDM [47]	36	6	Conformer	Accuracy 98.1%
		PAMAP2 [48]	9	18		Accuracy 99.7%
		UCI-HAR [49]	30	6		Accuracy 99.3%
	Jaramillo et al. [29], 2023	PAMAP2 [48]	9	5	Bi-LSTM	Accuracy 97.96%
	Challa et al. [30], 2023	PAMAP2 [48]	9	18	CNN + Bi-LSTM	**Accuracy 94.91%** **(Cross-subject)**
		UCI-HAR [49]	30	6		**Accuracy 97.16%** **(Cross-subject)**
		MHEALTH [50]	9	12		**Accuracy 99.25%** **(Cross-subject)**
	Pesenti et al. [26], 2023	Private dataset	12	5	LSTM	F1-Score 90.8%
	Hnoohom et al. [23], 2023	PPG-DaLiA [36]	15	8	PPG-NeXt	Accuracy 96.82% (10-fold)
		PPG-ACC [51]	7	3		Accuracy 99.11% (10-fold)
		Wrist PPG During Exercise [32]	8	4		Accuracy 98.18% (10-fold)
ECG	Arani et al. [44], 2021	PPG-DaLiA [36]	15	5	Random Forest	F1-Score 88.44% (10-fold)
						**F1-Score 60.34%** **(Leave-One-Subject-Out )**
	Almanifi et al. [22], 2022	Wrist PPG During Exercise [32]	8	4	Ensemble (Resnet50V2, MobileNetV2, Xception)	Accuracy 94.28%
	Hnoohom et al. [23], 2023	PPG-DaLiA [36]	15	8	PPG-NeXt	Accuracy 94.57% (10-fold)
		Wrist PPG During Exercise [32]	8	4		Accuracy 97.20% (10-fold)
PPG	Arani et al. [44], 2021	PPG-DaLiA [36]	15	5	Random Forest	F1-Score 62.65% (10-fold)
						**F1-Score 46.85%** **(Leave-One-Subject-Out )**
	Mahmud et al. [33], 2020	Wrist PPG During Exercise [32]	8	4	LSTM	Accuracy 72.1%
	Brophy et al. [31], 2018				Inception-v3	Accuracy 75.8%
	Almanifi et al. [22], 2022				Ensemble (Resnet50V2, MobileNetV2, Xception)	Accuracy 88.91%
	Hnoohom et al. [23], 2023	PPG-DaLiA [36]	15	8	PPG-NeXt	Accuracy 98.81% (10-fold)
		PPG-ACC [51]	7	3		Accuracy 92.22% (10-fold)
		Wrist PPG During Exercise [32]	8	4		Accuracy 91.65% (10-fold)
	Our Approach	Private dataset	40	5	CNN (proposed)	Accuracy 98.61% (5-fold)
						**Accuracy 95.14%** **(5-fold, Cross-subject)**
					PPG-NeXt	Accuracy 78.03% (5-fold)
						**Accuracy 70.33%** **(5-fold, Cross-subject)**
					LSTM	Accuracy 98.78% (5-fold)
						**Accuracy 83.63%** **(5-fold, Cross-subject)**
		PPG-DaLiA [36]	15	8	CNN (proposed)	**Accuracy 46.11%** **(5-fold, Cross-subject)**
				5		**Accuracy 60.77%** **(5-fold, Cross-subject)**
						**Accuracy 68.00%** **F1-Score 62.27%** **(Leave-One-Subject-Out)**
		PPG-ACC [51]	7	3		**Accuracy 78.67%** **(Leave-One-Subject-Out)**
		Wrist PPG During Exercise [32]	8	4		**Accuracy 85.87%** **(Leave-One-Subject-Out)**

Bold letters in the Performance column indicate results from the cross-subject approach.

## Data Availability

The datasets presented in this article are not readily available because of privacy and ethical concerns. The data supporting this study’s findings are available from the corresponding author upon reasonable request.

## Abbreviations

The following abbreviations are used in this manuscript:

HAR	human activity recognition
IMU	inertial measurement unit
EGM	electrogoniometers
EMGs	electromyograms
GSR	galvanic skin response
EDA	electrodermal activity
ECG	electrocardiogram
PPG	photoplethysmogram
ABP	ambulatory blood pressure
1D CNN	one-dimensional convolutional neural network
LSTM	long short-term memory
Leaky ReLU	leaky rectified linear unit
SD	standard deviation
CV	cross-validation
